# Expanded Insights Into Mechanisms of Gene Expression and Disease Related Disruptions

**DOI:** 10.3389/fmolb.2018.00101

**Published:** 2018-11-27

**Authors:** Moyra Smith, Pamela L. Flodman

**Affiliations:** Department of Pediatrics, University of California, Irvine, Irvine, CA, United States

**Keywords:** enhancers, transcription, domains, modifications malformations, enhancer, epigenetic, malformation, structural damage

## Abstract

Definitive molecular diagnoses in disorders apparently due to genetic or genomic defects are still lacking in a significant number of investigated cases, despite use of studies designed to discover defects in the protein coding regions of the genome. Increasingly studies are being designed to search for defects in the non-protein coding genome, and for alterations in gene expression. Here we review new insights into genomic elements involved in control of gene expression, including methods to analyze chromatin that is accessible for transcription factor binding, enhancers, chromatin looping, transcription, RNA binding proteins, and alternative splicing. We review new studies on levels of genome organization, including the occurrence of transcriptional domains and their boundary elements. Information is presented on specific malformation syndromes that arise due to structural genomic changes that impact the non-protein coding genome and sometimes impact specific transcriptional domains. We also review convergence of genome-wide association with studies of gene expression, discoveries related to expression quantitative trait loci and splicing quantitative trait loci and the relevance of these to specific complex common diseases. Aspects of epigenetic mechanisms and clinical applications of analyses of methylation signatures are also discussed.

## Introduction

During the last several decades much progress has been made in elucidating the nature of genomic and genetic defects that lead to congenital malformations and genetic disorders. Completion of the human genome project in 2003 and development of advanced techniques in genome sequencing have led to incorporation of DNA sequencing into clinical diagnostic protocols. The forms of sequencing most frequently carried out for clinical diagnostic purposes include targeted sequencing of specific genes and whole exome sequencing. There are reports indicating that exome sequencing approaches have led to successful diagnoses in 25–40% of patients with rare genetic diseases (Sawyer et al., 2016). There are now growing emphases on the non-protein coding genome and on regulatory elements and in addition to whole genome sequencing, various methods are being applied to analyze gene expression and the regulatory genome in clinical cases (Cummings et al., 2017). The Human Gene Mutation Data Base (Stenson et al., 2017), lists 3,801 mutations designated as regulatory.

Although still incompletely understood, recent research continues to expand insights into mechanisms of regulation of cellular differentiation and functions and insights into the regulation of developmental processes. Insights into regulatory processes are facilitated by analysis of genetic variations and through documentation of the effects of specific variants on development and function. In recent years it has become clear that variants in non-protein coding regions of the genome play key roles in determining specific human diseases and developmental disorders.

The goal of this review is to consider progress in delineating factors in gene regulation that need to be considered as we endeavor to elucidate the mechanisms involved in genetic and genomic diseases to facilitate molecular diagnoses, provide counseling and to improve management and treatment. This review includes information from 66 articles; 15 of the articles were listed as reviews. This review is organized into four main sections:

1. Analysis of genome elements that control gene expression and levels of genome organization.
2. Regulatory element disruptions and congenital malformations.
3. Polymorphisms that affect levels of gene expression; possible relevance to common disorders.
4. Epigenetic machinery, co-ordination of gene expression, epigenetic disorders, and diagnosis.

## Analysis of genomic elements that control gene expression and levels of genome organization

Consensus is emerging regarding the main gene regulatory elements. The main elements include gene promoters, enhancers, transcription factor binding sites, regions of open chromatin (DNAse1 hypersensitivity regions), and insulators. More recently evidence has emerged that chromatin looping plays an important role in bringing together enhancer elements and their specific promoters.

In a 2013 review entitled “Enhancers, Five Essential Questions,” Pennacchio, Bickmore, Dean, Nobrega, and Bejerano each individually presented views on how enhancers are recognized, how they function and how variants in enhancers impact evolution and disease occurrence (see Pennacchio et al., 2013).

Pennacchio defined enhancers as cis-acting sequences that increase gene transcription. He noted that recognition of enhancers is challenging because distances between enhancers and their specific target gene promoters are variable and may be extensive. Furthermore, enhancers may be downstream, upstream or within introns of genes. In addition, multiple different genes can be influenced by a specific enhancer. Pennacchio noted further that the sequence of specific enhancers is not clearly defined. He emphasized however that enhancer identification has been facilitated by recognition of highly conserved sequence elements that are present across different mammalian species. However, all such highly conserved sequences may not be enhancers. He noted that there are reports of some enhancers that are not highly conserved across species.

In addition to analysis of nucleotide sequences, active enhancers may be identified by specific histone modifications and epigenetic marks, such as H3K27ac and H3K4me and enhancers located in DNASE1 hypersensitivity regions. Enhancer sequence may correspond to P300 (EP300) binding sites. EP300 functions as a histone acetyl transferase and a chromatin modifier.

There is evidence for hundreds of thousands of enhancers in the human genome. Pennacchio noted the importance of studying enhancers and assessing their activity in different tissues.

Pennacchio et al. (2013) presented information on chromatin looping that leads to the physical association of enhancers and promoter regions of genes. She noted that in addition, promoter enhancer interaction may occur through supramolecular protein complexes. Specific proteins that bind to enhancers may also have affinity for specific promoters. One example of a protein that mediates interaction between an enhancer and a specific promoter is the Lim domain binding protein (LDB1).

Pennacchio et al. (2013) noted that DNA binding transcription factors could bind to both specific enhancers and promoters. Dean further noted evidence that general transcription factors and RNA polymerase II could be recruited to enhancers and promoters. There is also evidence that subunits of the Mediator complex and cohesin protein can localize to both promoters and enhancers. Dean also noted that RNA transcripts (eRNAs) may be generated from enhancer sequence elements. Active eRNAs are promoter bound and can therefore be isolated.

Pennacchio et al. (2013) reported that specific regulatory elements in human DNA are under evolutionary restraint. Pennacchio et al. (2013) reported that it was not yet known how many enhancers existed in the human genome and to what degree enhancers had undergone evolutionary changes.

### Additional functional elements in the non-protein coding genome and procedures for analysis

Bhatia and Kleinjan (2014) noted that there are different opinions regarding the proportion of the non-protein coding genome that is functional. The well-known genomic elements that impacts gene expression include promoters, enhancers, repressors, insulators, and chromatin organizing factors. Other key factors in gene transcription include the pre-initiation complex that binds RNA polymerase II and is required for mRNA synthesis. Additional regulatory elements likely exist.

Bhatia and Kleinjan defined procedures for identifying enhancers. These included DNAse1 hypersensitivity mapping and identification of transcription factor binding sites within the DNASE1 hypersensitivity sites. They noted that enhancers may be identified since they are often present in sequence elements with high evolutionary conservation. In addition, enhancers may be identified through demonstration of specific modifications in nearly histones, e.g., H3K3me1 or me3.

In 2015, Buenrostro et al. described ATAC-seq, a method to map and sequence accessible chromatin. Their method utilizes a unique transposase enzyme that detects and cleaves open chromatin and permits ligation of adapters at the cleaved ends so that DNA in these regions can then be sequenced. Utilization of the ATAC-seq methods enable identification of nucleotide elements present in open chromatin (Buenrostro et al., 2015).

Mathelier et al. (2015) considered transcription factor binding sites to be the core elements in cis-regulatory regions. They proposed that transcription factors load onto specific regions in accessible DNA and that this binding catalyzes recruitment of additional proteins. This may then bring about additional changes, including stabilization of transcription factor binding and epigenetic changes. They noted that by 2015 large numbers of regulatory regions had been identified in the human genome. Active regulatory regions can be determined by analysis of chromatin conformation, epigenetic modification, and transcription factor binding. Bound transcription factors can be identified by specific antibodies and antibodies can also be used to identify histone modification. Active chromatin regions can also be identified by chromatin conformation capture.

Mathelier et al. (2015) drew attention to CAGE technology (CAP analysis gene ends) that is based on analyses of sequences at the 5′ end of RNA transcripts. These analyses allow the determination of transcription start sites. In addition, RNAs transcribed from enhancer sequences (eRNAs), are captured through CAGE technologies. This then yields information on the enhancers that are associated with specific promoters.

In the Chip seq technology (chromatin immunoprecipitation and sequencing) DNA is cleaved and then treated with antibodies to transcription factors The DNA within the antibody bound fragments is then sequenced.

Machine learning computer approaches are also used to identify enhancer elements and promoters in genomic sequence data. Public databases are available with information on transcription factors and their DNA binding sites. The transcription factor binding site is most commonly 8–10 nucleotides in length.

### Enhancers: their structures and functions

Long et al. (2016) considered enhancers to be key factors in regulation of gene expression. They defined enhancers as non-protein coding cis regulatory elements, between 100 and 1,000 nucleotides in length, that participate in interactions with promoters of gene and that drive gene expression. There is also evidence that specific enhancers interact with other enhancers (Smemo et al., 2012).

Certain enhancers have cell specific functions. Long et al. proposed that divergence of enhancers played key roles in inter and intra-species phenotypic variation. They noted that there are very few deeply conserved enhancers across different species.

Long et al. noted that functional enhancers are composed of clusters of transcription factor binding sites. Coactivators are required by many enhancers for function. The co-activators are thought to act by modifying chromatin. Modifiers include histone methyltransferase, acetyltransferases, and chromatin modifiers. In addition, there are specific coactivators that interact with the promoter, e.g., Mediator protein complex.

The prediction that specific sequence elements function as active enhancers is based in part on the fact that active enhancers undergo specific histone modifications leading to the presence of Histone H3 lysine 4 monomethylation (H3K4me1). Further evidence of activation of expression includes the presence histone 3 lysine 27 acetylation (H3K27ac) at active enhancers and promoters and histone 3 lysine 4 trimethylation (H3K4me 3).

In addition, data has been gathered on the position of active enhancers and promoters in accessible DNA in the genome, e.g., DNAse hypersensitivity regions of the genome. Polymerase II binds to enhancers and RNA transcripts are generated from active enhancers.

For functional assessment of the enhancer activity of a specific DNA segment reporter assays are commonly used. The specific segments to be analyzed may be introduced into cells through plasmids or episomes, through microinjection or through retro viruses. In the latter case, the introduced sequence may be integrated into the genome (Chatterjee et al., 2016).

Another method to analyze enhancer activity involves disruption of the specific genomic segments containing the enhancer, e.g., through CRISPR Cas gene editing (Catarino and Stark, 2018).

Short et al. (2018) carried out sequence analyses of the non-protein coding genome in cases with neurodevelopmental defects. Their studies revealed an increased frequency in affected individuals of *de novo* mutation in enhancers and in sequences that regulate alternative splicing. Short et al. emphasized the urgent need for improved tools for functional assays in cells and in animal models to establish the functional impacts of specific regulatory and splicing mutations.

### Locus control regions

Taher et al. (2015) noted that regulatory elements in non-protein coding RNA may be present in the genome as discrete elements or they may be clustered in locus control regions (LCRs). One important LCR that is well-studied, is located upstream of the beta globin- like genes. Different elements in this LCR play roles in the differential expression of the different beta globin-like genes during development from embryonic life and into the newborn period.

### Gene transcription, splicing and alternative splicing

Gamazon and Stranger (2014) reviewed aspects of alternative splicing. They noted that in humans, studies have revealed that approximately 90% of genes undergo alternative splicing and that this contributes to RNA and protein complexity. RNA complexity derives from the use of alternative transcription start sites, alternative splicing, and use of alternative transcription termination sites and from RNA modification.

Key nucleotides in gene sequences that determine RNA splicing include 5 prime splice donor and 3 prime splice acceptor sites, and in introns, the splicing branch sites and polypyrimidine tracts. There is evidence that alternative splicing is influences by cis-acting exonic and intronic nucleotide elements including exonic splice enhancers and splice silencers. In addition, transacting factors and RNA binding proteins may impact splicing.

The most common forms of alternative splicing include exon skipping, use of alternative 5′ splice sites, use of alternative 3′ splice sites and intron retention, Gamazon and Stranger noted that intron retention is not common under physiological conditions.

There is evidence from evolutionary studies that alternative splicing leading to proteomic diversity occurs particularly in primates (Lindblad-Toh et al., 2011).

Gamazon and Stranger noted evidence that alternative splicing patterns occur during cell differentiation. Specific RNA binding proteins, such as the muscleblind proteins MBNL1 and MBNL2 were found to play important roles in remodeling of heart tissue during the post-natal period.

These authors emphasized that deep sequencing technologies continue to provide information on the extent of alternative splicing under physiological and pathological condition (Pajares et al., 2007). New therapeutic approaches to address pathological splicing defects include the use of antisense oligonucleotides.

Raj and Blencowe (2015) emphasized that the alternative splicing of gene transcripts results in the generation of different protein products from a specific gene, thus contributing to protein diversity. Alternative splicing is dependent on use of alternative splice sites in the nucleotide sequence of the gene and the primary transcript of that gene. Although alternative splicing is well-documented, the functional relevance of the alternative transcript isoforms has not always been defined.

These investigators reported that the cis-acting core splice site elements were insufficient to regulate alternative splicing and that specific transacting factors were necessary. The key transacting splice regulators recognized include polypyrimidine tract binding protein (PTBP1), NOVA 1 alternative splice regulator, RNA binding Fox homologs (RBFOX1, 2, and 3). Raj and Blencowe noted that these regulators primarily bind within 300 nucleotides of the splice site.

In addition, alternate isoforms of RNA binding splice regulators may manifest differences in their RNA binding sites. Other factors that impact splicing include heteronucleoproteins (hnRNPs) and serine arginine rich protein (SRRM3). Specific RNA binding proteins are particularly enriched in certain tissues.

Raj and Blencowe noted that tissue specific alternative splicing patterns arose from the combined activities of different splicing factors. Alternative splicing and its downstream effects result in the generation of different protein isoforms that may have different functions and different interactions with other proteins.

Raj and Blencowe reported that through application of high throughput genomic techniques, alternative splicing has been shown to play important roles in development of neural networks. Alternative splicing was shown to be particularly important in the developing nervous system in vertebrates. They reported that alternative splicing and alternative promoter usage led to extensive diversity of the protein products of the Neurexin and Neuroligin genes. Neurexin and neuroligin proteins play important roles in neurotransmission at synapses

Xiong et al. (2015) emphasized that in analyzing splicing it is important to consider the roles that RNA binding proteins play in influencing splicing. They noted that specific nucleotides have affinity for RNA binding proteins. The muscleblind-like proteins (MBNL) are important RNA binding proteins. In their comprehensive analyses, Xiong et al. identified 10,813 nucleotide variants that potentially impact splicing, many of these were found in introns.

Baralle and Giudice (2017) reviewed the role of alternative splicing in development and in differentiation of different tissues. They also presented information on the existences of splicing networks. In specific splicing networks there is apparent connectivity between splicing processes and timing of splicing of transcripts of different genes. This connectivity may in some cases be due to activity of specific RNA binding proteins.

Baralle and Giudice note that there is abundant evidence that specific human diseases result from mutations that impact 5′ or 3′ splice regulators or mutations that impact the spliceosome machinery. The spliceosome complex is composed of small nucleolar RNAs and multiple additional proteins. Baralle and Giudice note that key factors that influence the splicing process include splice site strength, cis regulatory expression of transacting proteins including RNA binding proteins and recognition of specific nucleotides in transcripts that are recognized by the spliceosome. Variation from the splice consensus sequences can lead to the generation of weak splice sites.

Development of microarrays imprinted with nucleotides corresponding to gene sequences has facilitated analyses of transcripts present in a particular cell type or tissue. Insight into co-ordination of splicing at a specific timepoint and in a specific developmental process has also been facilitated by increased availability of RNA sequencing technology.

Baralle and Giudice noted evidence that RNA binding proteins can contribute to co-ordination of alternative splicing patterns during development changes or in specific cell types. Recent studies have led to increased identification of specific target sites for RNA binding proteins. Another important factor studied relates to the degree of co-ordination in gene expression at a particular time.

Baralle and Giudice presented examples of alternative splicing that play key roles during development. In studies on embryonic brain cortex a significant percentage of specific genes were found to undergo alternative splicing so that isoform patterns differed from those found in adult brain. The levels of expression of the genes did not differ in adult and embryonic brain.

The specific genes that manifested different alternative splicing and different isoforms in embryonic brain vs. adult brain, included transcripts from genes encoding products that functioned in vesicle mediated transport, and transcripts from genes that encoded small GTPases. It is interesting to note that alternate splicing in some cases resulted in insertion of an exon that led to non-sense mediated decay of that transcript.

In delineating the specific factors that led to alternative splicing during brain development Baralle and Giudice referred to specific RNA binding proteins. These included pyrimidine binding tract proteins PTBP1 and PTBP2 and serine arginine repetitive protein SRRM4. Another important RNA binding protein involved in development is RBFOX1 that recognizes a (U)GCAUG sequence element in regulated exons or in flanking introns.

Baralle and Giudice reviewed information on alternative splicing and epigenetic factors. Alternative splicing of the enzyme histone lysine methyl transferase 2 (EHMT2 also known as KMT1C) that led to inclusion of exon 10 was shown to be important in brain development. Inclusion of this exon is thought to enhance nuclear localization of the enzyme and to increase methylation of histone H3. The transcripts of the enzyme KDM1 (histone lysine demethylase) were found to undergo alternative splicing and inclusion of exon 8a and this played important role in neurite morphogenesis. Changes in the levels of expression of KDM1 transcripts with exon 8a were also noted to impact neurite maturation.

Baralle and Giudice also noted that the extent of methylation of nucleotides within an exon impact inclusion of that exon in the final transcript of the gene.

They also noted that altered splice forms of specific proteins have been shown to play significant roles in specific neurological disorders. Altered splice forms of TAU transcripts leading to proteins with different number of repeat elements have been reported in Alzheimer disease.

A number of different genes have been shown to produce altered transcripts that impact heart development. Baralle and Giudice noted that these genes include TITIN. Proteins derived from different TITIN transcript isoforms differ in their degree of elasticity. Other proteins with alternate isoforms that are important in heart development include Muscleblind-like proteins MBNL1, DELF1, and RBFOX1 RBOX2.

Baralle and Giudice noted that alternative splicing of transcripts of the Fibronectin 1 gene was shown to impact the localization of the protein. Fibronectin 1 transcripts that included a specific exon referred to as the EDA exon were found to give rise to a protein that retained a cellular location. Fibronectin 1 transcripts from which the EDA exon were excluded gave rise to a soluble protein that was present in the plasma.

Baralle and Giudice concluded the review noting that ongoing research is concentrated on understanding the different functions and roles of different RNA isoforms and the proteins to which they give rise.

Rohacek et al. (2017) identified mutations in the epithelial splicing regulatory protein (ESRP1) that were associated with hearing loss. Through studies in Esrp1 negative mice, these investigators determined that deficiency of this splicing regulatory protein led to abnormal cochlear morphology and to abnormal hair cell differentiation. Through analysis of the transcriptome in Espr1 deficient mice they demonstrated aberrant splicing and impaired expression of a number of genes involved in cochlear development and auditory function.

### RNA binding proteins

Weyn-Vanhentenryck et al. (2014) undertook studies to define targets of RBFOX protein binding. Their studies led to identification of 1,059 genes with transcripts that undergo alternative splicing as a result of binding of RBFOX.

The specific functional categories of genes in which alternate splicing was impacted by RNA binding protein RBFOX included transcription factors, ion channels, ion transporters, neurotransmitter release factors, mitochondrial functions, and peroxisomal functions.

In these studies, the HITS-CLIP technology was used. In this technology high throughput sequencing of RNA follows isolation by cross linking immune-precipitation.

Weyn-Vanhentenryck et al. noted that findings of particular interest included evidence that 48 of the RBFOX binding genes that they identified had been implicated in autism though studies reported by other investigators. The genes CACNA1C (voltage gated calcium ion channels subunit A1C) and TSC2 Tuberous sclerosis 2) that have been implicated in autism, were found to be RBFOX targets.

Conboy (2017) reviewed the RBFOX family of RNA binding proteins and their critical roles in the regulation of developmental processes. RBFOX proteins bind at specific positions in the primary mRNA transcripts, including introns that flank exons that manifest tissue specific expression and 3′ untranslated regions.

There is also evidence that RBFOX proteins form part of a multi-protein complex that acts as a splicing regulator. This complex large assemblage of spice regulator (LASR) can bind at additional sites and binding is not restricted to the key RBFOX binding site.

RBFOX1 has been shown to play important roles in brain development. Knockout of RBFOX1 or RBFOX2 in the developing brain in mice was shown to lead to neurological defects particularly in the cerebellum, to movement disorders and to epilepsy. Conboy also documented reports of RBFOX1 deletions in humans and association of deletions that impacted the promoter and or exons that led to neurodevelopmental defects. RBFOX1 and RBFOX2 deletions have also been reported in association with congenital heart disease.

### Transcription domains

Farrell et al. (2002), Ghirlando et al. (2012) first described transcription domains that were flanked by nucleotide sequences that bound the CCCTC binding transcription factor designated CTCF. Dixon et al. (2012) referred to the CTCF binding domains within the genome as topologically associated domains (TADs).

Spielmann and Mundlos (2016) reviewed regulatory elements in the non-protein coding genome and the roles of variants in these elements in causation of human disease. They noted that the promoter regions of genes are usually located <1 kilobase from transcription start sites. Enhancers may be located relatively close to promoters but are often located at great distances from promoters. Both promoters and enhancers serve as docking sites for general transcription factors. Enhancer sequences are sometimes primed by pioneering transcription factors that subsequently serve as docking platforms for general transcription factors.

Looping of chromatin may serve to bridge distances between promoters and enhancers and specific proteins facilitate connection of enhancers to promoter regions. These proteins include Mediator, CTCF (CCCTC binding factor), and Cohesins.

Specific techniques, including chromosome conformation capture (3C and High C) have been developed to isolate and analyze regulatory elements and genomic sequences with which they interact.

Such techniques have led to the identifications of TADs. Specific TADs are prevented from contact with neighboring domains by specific boundary elements that include CTCF and cohesin. CTCF is essential for establishing TADs but it also has other functions. Spielmann and Mundlos emphasized that TADs play key roles in the control of gene expression. There is growing evidence that disruption of TADs through structural genomic changes constitute important disease mechanisms (Lupiáñez et al., 2015; Kaiser and Semple, 2017).

### Different levels of genomic organization

Hansen et al. (2018) reported that different levels of organization exist within the genome. One form of organization involves the position of chromosomes within the nucleus, leading to regions designated as chromosome territories. In addition, within the genome there are regions that are transcriptionally active and other regions that are inactive. Hansen et al. referred to these as the A active compartment and B, the inactive compartment. Active chromosome regions are associated with active chromatin and they form epigenomic compartments that tend to be located away from the nuclear envelope. The inactive chromatin compartments tend to be located near the nuclear envelope and to constitute the lamina associated chromatin and chromosome domains.

Hansen et al. noted that there is evidence that TADs constitute functional domains in which specific enhancer promoter contacts can activate gene expression. Numerous studies have demonstrated the importance of the CTCF protein and cohesin protein in defining TAD boundaries. CTCF is an 11 Zinc finger DNA binding protein, the CTCF protein forms loops. Cohesin is a multiprotein complex that adopts a ring structure. The cohesin protein complex is important not only at TAD boundaries, it is also important in linking sister chromatids following replication of chromosomal DNA. Components of cohesin include SMC1 and SMC3 (SMC structural maintenance of chromosomes), RAD21 (SCC) cohesin component. The protein NIPBL genes encodes a protein that is essential for loading of cohesin. The NIPBL gene is defective in Cornelia de Lange syndrome (CdLS). Newkirk et al. (2017) reported a global reduction in loading of cohesin onto CTCF binding sites in cases with NIPBL mutations and CdLS and there was evidence of dysregulated gene expression.

Hansen et al. noted that the high C chromatin conformation analysis technique provides data that is the average gathered from a large cell population. They used live cell imaging techniques and single cell analysis. They used live cell imaging to investigate TADs at the single cell level and they analyzed TAD formation over times.

These investigators reported that some TADs may have a configuration where smaller TADs are nested within larger TADs. Another important question that needs to be addressed is whether TADs and chromatin loops are stable or whether they are dynamic structures. Their studies led them to conclude that cohesin mediated loops were dynamic structures and that these structures facilitate dynamic and repeated promoter interactions in cells.

### CTCF and cell differentiation

Arzate-Mejía et al. (2018) reviewed evidence that CTCF synthesis is regulated during development and that it plays roles in cell differentiation. They reported that if CTCF is depleted in early embryos, they do not implant. There are reports that CTCF levels are high in embryonic brain and that depletion of CTCF can lead to defects in brain development. CTCF was also found to be important for survival of post-mitotic neurons. Removal of CTCF during limb development in the mouse leads to altered gene expression and to limb truncation.

They proposed that control of CTCF synthesis, along with control of synthesis of specific transcription factors led to chromatin looping that in turn led to specific patterns of gene expression.

Arzate-Mejia et al. described the specific domains in CTCF protein. Domains 3 to 7 constitute the central zinc finger domains that bind to a 15 base pair motif in DNA. Other Zn finger domains in CTCF can bind to other nucleotide sequences in DNA. The N- and C-terminal domains of CTCF protein bind to other proteins. There is evidence that DNA methylation can impact CTCF binding. In addition, promoter enhancer interactions can be impacted by proteins other than CTCF.

There is also evidence that perturbation of specific CTCF binding sites can impact gene expression and differentiation. Arzate-Mejia et al. noted that gene expression and development can also be disrupted through changes that impair the interaction of CTCF with specific transcription factors or disruptions of the interaction of CTCF with cohesin and impairments of appropriate chromatin looping.

### Mechanisms underlying position effect

The term position effect was often employed in the past to explain the consequences of structural genomic defects. Spielmann et al. (2018) noted that given recent advances in knowledge about enhancers and TADs and the importance of TAD boundaries, position effects can now be better understood. The term position effect was applied to classical aniridia occurring in patients with chromosome defects close to the PAX6 gene.

## Regulatory element disruptions and congenital malformations

### PAX 6 and aniridia

Classical aniridia is a developmental eye defect associated with absence of the iris and panocular defects. In some cases, this disorder is due to mutations in the protein coding sequence of the PAX6 gene on chromosome 11p13. Fantes et al. (1995) described pedigrees with aniridia patients in whom no mutations in the PAX6 coding sequence were detected; however, these patients were found to have chromosome rearrangement with breakpoints 85 kb distal to the 3′ end of PAX6.

Lauderdale et al. (2000) reported two cases with aniridia and intact PAX6 protein coding sequence but with *de novo* deletions 11 kb from the 3′ end of PAX6. They suggested that a sequence element in that region was required for PAX6 expression.

The eye disease Aniridia is due in some cases to defects in the PAX6 gene defect associated with haploinsufficiency. Aniridia has in some cases been shown to be due to defects in a sequence element located in an intron of the ELP4 gene that maps close to PAX6. Regulation of expression of PAX6 requires a number of different regulatory elements (Crolla and van Heyningen, 2002).

Bhatia et al. (2013) reported results of studies in cases of aniridia with intact PAX6 protein coding sequence but with disruption of an ultra-conserved cis-regulatory element located 150 kb downstream from PAX6. They established that the disrupted element encoded an enhancer element.

### SHH sonic hedgehog gene (SHH)

The hedgehog gene HH was first discovered in Drosophila by Nüsslein-Volhard and Wieschaus (1980) and subsequent studies in vertebrates revealed the presence of 3 hedgehog genes designated Sonic Hedgehog (SHH), Indian Hedgehog (IHH), and Desert hedgehog (DHH).

There are growing numbers of reports on the roles of regulatory element mutations in causation of specific developmental defects. These include reports of mutations in a regulatory element one megabase distant from the human SHH (sonic hedgehog gene) that lead to pre-axial polydactyly (Lettice et al., 2003).

SHH gene expression has also been found to be disrupted in specific cases with abnormal limb development. The ZRS regulatory element is particularly important in control of SHH expression in limb developments. Mutations in the ZRS elements have been shown to lead to limb malformations. Specific limb malformations, including triphalangeal thumb and syndactyly can result from duplications of ZRS that lead to increases SHH activity. Deletion of ZRS can result in truncation of limbs and absence of feet. Microduplications of the ZRS were reported to lead to aberrant expression of SHH that resulted in polydactyly, syndactyly, and sometime duplications of fibulae (Lohan et al., 2014).

Roessler et al. (1997) reported that cytogenetic abnormalities in chromosome 7q36 that led to loss of SHH2 resulted in holoprosencephaly. They also reported that specific mutations in SHH led to holoprosencephaly. This disorder disrupts development of midline structures of the brain and face.

Anderson and Hill (2014) in studies on mouse reported that three regulatory elements located upstream of SHH, MACS1 MFCS4 and MRCS1 control expression of SHH in the epithelial linings. MACS1 impacts epithelial development in trachea, lungs and urogenital system. MFCS4 impacts development of epithelium of the soft palate and epiglottis; MRCS1 impacts development of teeth, hard palate and tongue epithelium and of the pharynx, lung and digestive tract.

### Recent reports related to SHH

Xavier et al. (2016) reported that during early development SHH is produced in the neuroectoderm of the forebrain and in the pharyngeal endoderm, SHH signal is also required for division of the eye-field and the forebrain.

In a review published in 2016, Rimkus et al. (2016) noted that SHH signaling plays roles in cell differentiation, cell proliferation and polarity not only during development but also in tumorigenesis and cancer. They also focused attention on the downstream effectors of SHH including Smoothened (SMO) and the GLI family of transcription factors.

Rimkus et al. noted that SHH is the most active hedgehog gene and it continues to be expressed in adult life, Furthermore, hyperactivation of the SHH signaling pathway has been found in many tumors.

The SHH protein undergoes modification by cholesterol prior to its release from cells. Cholesterol is added to both the N and C terminals of the proteins. On its release from cells SHH binds to the PTCH (Patched) protein and this releases the attachment of PTCH1 to SMO and releases PTCH from the cilia membrane. PTCH1 then serves as a receptor for SHH. The released SMO activates expression of GLI transcription factors. GLI transcription factors then enter the nucleus and activate gene expression.

### Copy number variants in the non-protein coding genome that can alter regulation and lead to defects

Flöttmann et al. (2017) reviewed copy number variants in non-protein coding regions of the genome that were found to be associated with limb malformations. Their studies included 340 individuals with isolated limb malformations and in 35 cases they identified copy number variants that were potentially causative. Of these 35 patients, 16 had CNVs that did not include a specific disease gene; 3 of these patients had a deletion that impacted a known enhancer of the DLX5/6 genes, the distal-less homeobox genes, known to play roles in bone development.

Eleven of the 35 patients had duplications of enhancer elements that led to gain of function either in the SHH (sonic hedgehog gene) or in FGF8 gene (fibroblast growth factor gene 8). These genes encode proteins that play roles in morphogenesis.

Two of the 35 patients had CNVs that led to disruption of a boundary element (TAD) of the PAX3 gene and expression of this gene was altered. PAX3 (Paired box 3 gene) encodes a transcription factor that plays key roles in development.

Flöttmann et al. reported additional novel CNVs that were likely causative of defects. These included a 440 kb microdeletion in chromosome 2q31 in a regulatory region of the HOX D (homeobox) gene cluster. Genes in this cluster are involved in morphogenesis. In another patient a duplication was found that encompassed and enhancer of the TBX 5 gene that encodes a transcription factor involved in developmental processes.

### Structural genomic variation in specific human diseases

Spielmann et al. (2018) reviewed structural genomic variation in specific human diseases. They noted that clinical interpretations of the pathologic consequences of structural variants remains unsatisfactory. Three aspects of structural genomic variation need to be taken into account. Structural variants may lead to disruption of gene dosage, they may impact gene regulatory elements in non-protein coding regions of the genome, and they may lead to disruption of chromatin domains.

Spielmann et al. noted that microarray techniques involving hybridization of genomic DNA are often used to identify copy number variants; however, these methodologies are usually of low resolution. Genomic sequencing that employs short read technologies fails to detect breakpoints and rearrangements that impact repetitive regions. Long sequencing techniques and single molecule sequencing can provide more accurate information. Currently several large-scale projects are underway to develop structural variant maps of the human genome, e.g., the 100,000-genome project.

Spielmann et al. noted that there is growing evidence that even well-studied structural genomic variants, e.g., 15q13.3 microdeletion show reduced penetrance in some cases and the clinical manifestations in affected patients vary.

Several structural variants have been shown to lead to pathology through alterations of dosage of protein coding genes. Spielmann et al. noted that the 22q11.2 deletion likely leads to cardio-vascular defects through deletion and reduced dosage of TBX1, a transcription factor. The 17q21.1 microdeletion reduces expression of KANSI 1 that encodes subunits of an enzyme involved in histone lysine acetylation. This deletion is associated with craniosynostosis in some patients, some patients may manifest epilepsy and others may manifest cognitive impairments. Manifestations of the Smith–Magenis syndrome are thought to be primarily due to deletion of the RAI1 gene (retinoic acid induced) on 17p11.2. This gene is expressed primarily in neuronal tissues. Smith–Magenis syndrome may be associated with autism. Chromosome 2q13.1 deletion most likely leads to autism and intellectual disability because MBD5 (methyl CpG binding domain protein 5) is deleted.

Spielmann et al. (2018) emphasized the importance of taking into account information on chromosome folding, regulatory elements, and TADs when considering the effects of structural chromosome variants.

Structural variants that impact 3D organization of the genome include copy number neutral variants including inversions, insertions, and translocations. There are also reports of examples of developmental defects resulting from disruption of TAD boundary elements.

### SRY SOX9 and developmental defects

Koopman et al. (1991) reported that the SRY gene (sex determining region on Y) encodes a protein that is expressed in the embryo and that triggers development of Sertoli cells that are essential for testis differentiation. The gene that encodes the transcription factor SOX9 was subsequently shown to be the main SRY target. Jo et al. (2014) demonstrated that SOX9 expression occurs in many different cell types. Symon and Harley (2017) reported that the SOX9 regulatory region extends for 2 megabases 5′ to the protein coding sequence.

Baetens et al. (2017) reviewed variations in the genome that were associated with disorders of sex development (DSD) They noted that earlier studies had revealed gene changes that encode in several different transcription factors. These included mutations in:

SRY1 Sex determining region on Y chromosome, encodes a transcription factor.

SOX9 SRY-box DNA binding protein, recognizes the sequence CCTTGAG.

NR5A, nuclear receptor 5A, encodes a DNA binding protein zinc finger transcription factor.

FOXL2 Forkhead box transcription factor.

Baetens et al. noted that elements that regulate expression of SOX3 gene (SRYBox3) on Xq27.1 have also been identified based on cytogenetic studies. In addition, genomic regions upstream of the DMRT1 gene (DM domain containing transcription factor) on chromosome 9p24.3 have been found to harbor regulatory elements that impact expression of that gene. Deletions in these upstream regions have been found to lead to abnormalities of sexual development.

However, in many cases of disorders of sexual development disorders defects in protein coding genes have not been identified. More recently detailed studies have been carried out in regulatory regions of the genome and have led to identification of genomic alteration involved in the causation of disorders.

Gonen et al. (2018) reported that one specific enhancer elements in the SOX9 upstream regulatory region plays a key role in SOX9 expression and in male gonad differentiation. Through studies in the mouse they determined that the Enh13 enhancer was essential for initiation of testis development. They reported that Enh13 sequence is highly conserved in mammalian species. The Enh13 enhancer elements is 557 nucleotides in length.

### Defects in the SOX9 gene region in a range of congenital defects

Structural variants in the genome region surrounding the SOX9 gene have yielded results of particular interest. Studies of patients with specific congenital abnormalities and defects in the genomic regions flanking SOX9 led to discovery of deletions of different specific genomic segments, each associated with particular phenotypic abnormalities.

Defects in the genomic region 1,230–1,036 kb upstream of SOX9 were associated with Pierre Robin sequence, a congenital facial, jaw, and upper airway anomaly.

Defects in the genomic region between 517 and 595 kb upstream of SOX9 were associated with a mild form of campomelic dysplasia a congenital defect associated with limb abnormalities and defects in development of genitalia

Duplication of a region between 517 and 595 kb upstream of SOX9 led to disorders of sexual development in individuals with 46XX genotype

Deletion of a region 607–16,396 kb upstream of SOX9 led to abnormalities of sexual development in individuals with the 46XY genotype.

In addition, duplication of a region further upstream of the SOX9 gene toward the KCNJ16 potassium channel gene was associated with Cook syndrome, in which congenital limb abnormalities and nail defects occur.

Franke et al. (2016) reported use of chromatin conformation capture methods to identify specific boundary elements. They proposed that different regions upstream of SOX9 are separated by boundary elements and that the different regions represent TADs.

### Transcriptome analysis in diagnosis of monogenic diseases

Kremer et al. (2017) reported use of RNA sequencing to establish causative diagnoses in patients with clinical evidence of mitochondrial functional disorders. They noted that RNA analyses can yield information on RNA abundance, nucleotide variants, allele specific gene expression, and information on the relative abundance of specific splice isoforms.

Kremer et al. considered genetic causes of aberrant RNA expression levels. These include variants in the protein coding segments of a specific gene, rare variants in gene promoters and variants in regulatory elements. Monoallelic expression can occur when one allele is silenced and an allele may be silenced due to a regulatory element.

The patients studied by Kremer et al. included 48 patients with suspected mitochondrial disease where exome sequencing had failed to yield genetic diagnosis. Abnormalities detected in these patients on the basis of RNA sequencing included evidence of down-regulation of RNA for mitochondrial protein for translocase of inner mitochondrial membrane domain containing (TIMMDC1) that maps to chromosome 3q13.33, in two cases. Abnormal splicing was identified in TIMMDC1. The abnormality in TIMMDC1 Included finding an RNA isoform with a new exon derived from genomic sequence in intron 5 of the gene. Abnormal splicing was also found in the caseinolytic mitochondrial matric peptidase proteolytic (CLPP) RNA in one case. The CLPP gene maps to chromosome 19p13.3. The abnormality found in CLPP RNA was found to involve exon skipping leading to truncated transcript. In one case there was down-regulation of microsomal glutathione S transferase 1 (MGST1) that maps to chromosome 12p13.3.

Monoallelic expression leading to decreased gene expression of aldehyde dehydrogenase ALDH18A1 was found in one patient. This led to very low levels of ALDH18A protein, a bifunctional mitochondrial protein encoded by a gene that maps to chromosome 10q24.1.

The RNA studies described by Kremer provided diagnoses in 5 of the 48 patients in whom exome sequencing had failed to detect abnormalities. Kremer et al. emphasized that prediction of splicing defects on the basis of exome sequencing is not straight forward since all of the elements and factors involved in splicing have not been clearly defined.

## Polymorphisms that affect levels of gene expression; possible relevance to common disorders

In 2017, the Genotype Tissue Expression Consortium (GTEx) published data on nucleotide variants that impact levels of gene expression. The specific variants analyzed that impacted expression were located in promoters, enhancers, and repressors. Elements that altered quantitative levels of expression of a gene on one member of a specific chromosome pair were referred to as cis quantitative trait loci (Cis QTLs). The cis QTLs were found to usually be located in close proximity to the gene that showed altered levels of expression. Trans QTLs can alter expression of genes on more than one chromosome and are located at some distance from genes they regulate (Ward and Gilad, 2017). Gene expression can also be altered through RNA editing.

### Genome-wide association studies (GWAS) and eQTLs (expression quantitative trait loci)

Specific regions of the genome associated with specific diseases were identified through genome-wide association studies (GWAS). For the majority of GWAS associations the precise molecular mechanisms leading to disease have not been identified. One of the difficulties encountered in attempting to identify specific sequence variants within loci found on GWAS, is the phenomenon of linkage disequilibrium that leads to co-inheritance of a series of variants the map within a defined genomic segment.

Through studies carried out by the Battle and Montgomery (2014) and GTEx Consortium (2015, 2017) levels of gene expression in different tissues were documented and resources have been developed that facilitate identification of expression quantitative trait loci (eQTLs). Other molecular data that has been gathered in this project includes information on transcription factor binding sites, positions of DNA methylation, histone modification, chromatin accessibility, and alternative splicing.

The GTEx consortium reported that information on methylation QTLs have revealed relationships between genetic variants, methylation variants and gene expression. They noted further that transcription and translation variants ultimately lead to variations in protein levels.

In 2017, progress was reported in the GTEx project that seeks to facilitate analysis of the biological impact of disease related variants. The GTEx project has involved analyses of transcription in a number of different tissues from specific individuals. Approximately 45 different tissue sites were sampled from each of 237 donors in the pilot project. Patterns of gene expression were analyzed and genomic sequence was obtained on each individual. The GTEx project also documented eQTLs. Comprehensive studies revealed that of the 22,286 genes studied, 10,030 had eQTLs. The eQTLs tended to show an upstream bias in position. At least 68% of the eQTLs they identified had been identified in prior studies on blood samples. The GTEx studies revealed that more than 50% of the eQTLs operated in multiple tissues.

### Splicing QTLs (sQTLs) splicing quantitative trait loci

RNA sequencing (RNA seq) enables analysis of levels of expression of different transcript isoforms. In the GTEx project factors that lead to differential inclusion of particular exons in RNA transcripts were referred to as sQTLs. The splice variants detected were variants that led to exon skipping in 80% of cases.

In Li et al. (2016), investigated the effects of variants on pre-mRNA splicing. They identified sQTLs that impacted 2,313 genes. The sQTLs they identified were located within genes and primarily in introns.

### Interrogating the functional effects of disease associated loci found on GWAS

Hauberg et al. (2017) carried out studies to determine the possible relationships loci found on GWAS studies with expression QTLs and associated elements. Their studies involved different tissue types and results of their studies led to insights on how common trait loci alter gene expression.

They noted that eQTL data base resources used in the GTEx project, included STARNET (Studies of RNA expression on disease relevant tissues) and CMC (Commonmind Consortium database). They noted that, in addition, several international projects have led to the establishment of databases with comprehensive information in genome-wide association variants in specific diseases.

Hauberg et al. used specific analysis tools to test for joint association of GWAS and eQTL data. In addition, they carried out analysis of gene expression in different tissues. Their studies revealed that association of GWAS markers and eQTLs and gene expression were more significant in some tissues than on others.

It is important to note that a significant disease associated allele at an eQTL locus can be associated with increased gene expression while another significant disease associated allele at an eQTL locus can be associated with decreased gene expression. Furthermore, some eQTLs were found to impact gene expression primarily in one tissue, while other eQTLs impacted expression in more than one tissue. Hauberg et al., emphasized that the relevant eQTL for a specific disease trait often acted in a specific set of tissues. In the STARNET project that identified GWAS loci for coronary heart disease and abnormalities in lipids, eQTL loci were found to be more associated with abnormal gene expression in arterial or adipose tissue samples.

Hauberg et al. reported that their studies revealed that the gene with altered expression was not always the gene closest to the relevant eQTL.

### Analysis of transcribed enhancers in schizophrenia cases and controls

Hauberg et al. (2018) undertook analyses of RNA transcribed from enhancer sequences, described as eRNAs and an analysis of gene expression together with sequencing of transposase accessible chromatin. The samples they analyzed included total RNA isolated from the dorsolateral pre-frontal cortices of brains derived from 258 schizophrenia patients and 279 controls. They selected this brain region since it is known to have particular relevance to cognitive and psychotic symptoms. Genotype information was also available on these patients.

This study led to identification of variants in 927 enhancers that Hauberg et al. designated as enhancer expression quantitative trait loci (eeQTLs). In addition, 118 enhancer RNAs were found to be differentially transcribed in schizophrenia cases and these different eeQTLs correlated with altered findings in expression studies.

Genes that manifested altered expression converged on a number of neurologically relevant biological pathways. Hauberg et al. emphasized convergence on the Roundabout receptor (ROBO) pathway that is involved in the cytoskeletal remodeling necessary for axonal and dendritic branching.

## Epigenetic machinery, co-ordination of gene expression, and epigenetic disorders

### Co-ordination osf gene expression and metabolic state

van der Knaap and Verrijzer (2016) reviewed aspects of metabolism and metabolic enzymes in the control of gene expression. They emphasized that the co-ordination of metabolic state and gene expression were essential for cell growth, differentiation and homeostasis. They noted that modifications of chromatin that play critical roles in gene expression, depend on metabolites and cofactors.

In considering metabolism, van der Knaap and Verrijzer documented key metabolic processes involved in catabolism and in anabolism. These investigators emphasized that almost all enzymes that modify chromatin utilize metabolites derived from intermediary metabolism. Important among these are acetyl donors and acetyl-coenzyme A that are utilized by histone lysine acetyl transferase to acetylate histone. Generation of acetyl-coenzyme A takes place at several steps in metabolism and cellular levels of acetyl-coenzyme A fluctuate depending on nutrient availability. There is also evidence that acetylation of histones at specific positions, creates binding sites for regulators of chromatin structure, such as bromodomain containing regulators.

S-adenosyl methionine serves as donor of methyl groups used both for the methylation of histone and for the methylation of DNA. In humans adequate functioning of one carbon metabolism that involves tetrahydrofolate is essential for adequate synthesis of S-adenosyl methionine.

Sharma and Rando (2017) reviewed metabolism and epigenetic modifications. They noted that methylation of DNA requires S-adenosyl methionine. The TET enzymes that carry out demethylation of cytosine involves preliminary conversion of methyl cytosine and these reactions require alpha ketoglutarate and Vitamin C. Chromatin remodeling and alteration of nucleosome positions requires ATP dependent enzymes.

Histone acetylation requires the presence of acetyl-coenzyme A. Sharma and Rondo noted that specific histone deacetylases that remove acetyl groups from histones, require nicotinamide adenine dinucleotide (NAD) as co-factor.

Histone methylation requires S-adenosyl methionine and iron. Specific histone demethylases require flavin adenine dinucleotide (FAD).

Sharma and Rondo concluded that given the important roles of metabolites in epigenetic processes, it will be important to continue to investigate the impact of alterations in nutrient intake on epigenetic modifications during embryonic life and beyond.

### Approaches to analyze defects of epigenetic machinery

Disorders of epigenetic machinery arise due to defects in chromatin, writers, erasers, or remodelers (Borrelli et al., 2008). Bjornsson (2015) reviewed these disorders. Clinically they are often associated with neurodevelopmental dysfunction, however other physical abnormalities are often present. Furthermore, overlapping clinical manifestations often occur in the different disorders due to disruptions or mutations in different genes that encode components of the epigenetic machinery.

### Key components of the epigenetic machinery

Cytosine methylation, particularly of CpG dinucleotides, involves the activity of methyltransferases DNMT1, DNMT3A, DNMT3B. Removal of methyl groups from cytosine involves conversion of methyl cytosine to intermediates and the activity of the TET enzymes, methyl cytosine dioxygenases, TET1, TET2, and TET3. Specific readers of cytosine methylation include methyl CpG-binding domain proteins MBD1, MBD2, and MECP2.

Important modifications of histones include mono, di, and trimethylation and addition of acetylation to histone tails. The specific form of histone modified most frequently histone H3 and the specific histone residue modified are important in determining the effects of modification. Important readers of histone modifications include chromodomain and bromodomain protein complexes.

### Mendelian disorders of epigenetic machinery

Bjornsson (2015) documented 44 Mendelian disorders due to defects in the epigenetic machinery. He noted that 93% of these disorders were associated with neurological dysfunctions. Other key manifestations included growth abnormalities, either growth deficiency in some disorders and overgrowth in other disorders. In addition, limb and nail abnormalities were common and immune dysfunction occurred in some forms.

Bjornsson subclassified disorders into 4 categories, disorders due to defects in erasers, in writers, in readers or in modifiers and listed the genes known to be defective in each. Eighteen different genes that encode chromatin writers were found to harbor mutations that led to Mendelian disorders. Mutations in chromatin modifiers were found in 13 different disorders. Seven different disorders were due to defects in eraser encoding genes, and six different disorders were known to be due to defects in genes that encode readers.

Bjornsson reported that the majority of disorders of the epigenetic machinery are autosomal dominant disorders, only 15% of disorders were autosomal recessive disorders. Furthermore, most of the disorders arose from haploinsufficiency, including nucleotide changes leading to loss of function, or deletion mutations. He noted further that the number of different organ systems involved in diseases of the epigenetic machinery was significantly larger than the number of organ systems involved in most other Mendelian disorders and that it seemed likely that in diseases of the epigenetic machinery a number of different target genes were impacted (see Supplementary Material).

### Diagnostic utility of defining genome-wide DNA methylation signatures

Choufani et al. (2015) reported results of studies designed to generate a genome-wide DNA methylation signature in blood cell DNA from patients with Sotos syndrome. This syndrome is due to mutations in the NSD1 gene that encodes nuclear receptor SET domain protein. Choufani et al. reported that using the Illumina Infinium Human Methylation 450 bead chip over 28,000 CpG sites could be examined. Microarray data were analyzed to identify the intensity of signal and the evidence of loss of signal at specific sites.

The NSD^+/−^ patients 7,038 CpG sites revealed loss of DNA methylation signals and 47 sites showed increased methylation. The investigators then developed a predictive model that classified patients on the basis of their CpG methylation patterns.

Importantly results of their study revealed that CpG methylation patterns facilitated distinction of patients with pathogenic mutations in NSD1 from patients with nucleotide variants in NSD1 that were classified as of uncertain significance. In addition, analyses of patterns of methylation enabled distinction of Sotos syndrome from patients with a clinically similar disorder Weaver syndrome also due to NSD1 mutations.

Choufani et al. concluded that the CpG methylation signatures could act as a functional test for Sotos syndrome.

Butcher et al. (2017) undertook studies on DNA methylation signatures on patients with clinical diagnosis of CHARGE syndrome and patients with Kabuki syndrome. CHARGE syndrome is due to pathogenic mutations in the CHD7 gene (chromosome domain helicase DNA binding protein 7). Kabuki syndrome is due to pathogenic mutation in KMT2D (lysine methyl transferase 2D). Butcher et al., noted that there is extensive clinical overlap in manifestations in these patients, particularly in early life. In addition, in some cases with clinical manifestation indicative of these syndromes exome sequencing data reported only nucleotide variants that were classified as of unknown significance.

For their study Butcher et al. isolated DNA from patient blood cells. DNA was then treated with bisulfite and subsequently hybridized to Illumina Infinium Human Methylation 450 bead chip arrays.

Butcher et al. reported that they identified unique methylation signatures in patients with pathogenic CHD7 mutations and different unique methylation signatures in patients with pathogenic KMT2D mutations. The two disorders shared findings of decreased methylation in HOXA5. Importantly methylation signatures were negative in patients who had been found on exome sequencing to have nucleotide variants of uncertain significance.

Schenkel et al. (2017) reported identification of a unique epigenetic signature in patients with alpha thalassemia mental retardation syndrome (ATRX). This disorder is due to mutation in a gene that maps to the X chromosome and encodes a chromatin remodeling protein. The Schenkel study involved studies on 18 ATRX patients and on 2010 controls. Studies were carried out DNA from blood cells. DNA was bisulfite treated and then hybridized to the Illumina Infinium Human Methylation bead chip arrays. Intensity values for hybridization were determined and analyzed using Illumina genome Studio Software.

Analyses revealed marked asymmetry in signals from patients and those from controls. In the ATRX patient samples 16 genomic regions were found to have increased methylation compared with controls and three genomic regions showed decreased methylation in patients vs. controls.

Schenkel et al. evaluated the biological functions of genes that yielded altered methylation patterns in the ATRX patients. They determined that there was over-representation of genes involved in biosynthetic processes, nucleic acid metabolism, and methylation processes. They proposed that the ATRX mutation resulted in transcriptional dysregulation of several genes and that dysregulation led to ATRX neurodevelopmental problems.

## Conclusion

Significant progress has been made in recent decades in elucidating the different mechanisms of gene expression and the important roles of elements in the non-protein coding genome in regulation of gene expression. More comprehensive information has been gathered regarding epigenetic modifications of DNA and chromatin. In addition, there are new insights into the different levels of genome organization, including the demonstration of specific domains with boundary elements and the importance of chromatin looping mechanisms in control of gene expression.

It is important that these new insights be taken into account as we search for causes of genetic and genomic diseases.

## Author contributions

All authors listed have made a substantial, direct and intellectual contribution to the work, and approved it for publication.

### Conflict of interest statement

The authors declare that the research was conducted in the absence of any commercial or financial relationships that could be construed as a potential conflict of interest.
