# Supplementary material for: Expanded Insights Into Mechanisms of Gene Expression and Disease Related Disruptions
Source: Front Mol Biosci. 2018 Nov 27;5:101. doi: 10.3389/fmolb.2018.00101 (PMC6277798; doi:10.3389/fmolb.2018.00101)
Supplement: Supplementary file 1 [file Table_1.DOCX]

**Malformation syndromes shown to be due to Regulatory Gene Defects**

| **Gene**  PAX6  SHH  DLX5/DLX6  SRY1, SOX9, NR5A, FOXL2  SOX9  TIMMDC1, ALDH18A1, MGST1 | **Malformations. Functional defects**  Aniridia  Holoprosencephaly, Pre-axial polydactyly. Oropharyngeal defects  Limb defects  Disorders of Sexual development  Facial defects, upper airway defects, Limb abnormalities  Impaired mitochondrial function |
| --- | --- |
| **Gene**  NSD1  CHD7  KMT2D  ATRX | **Altered epigenetic modifications at genomic sites distant from defective gene**  SOTOS syndrome  CHARGE syndrome  KABUKI syndrome  Alpha-thalassemia mental retardation Syndrome |
